# Molecular basis of atypicality of bupropion inferred from its receptor engagement in nervous system tissues

**DOI:** 10.1007/s00213-018-4958-9

**Published:** 2018-07-01

**Authors:** Eric J. Kim, Klara Felsovalyi, Lauren M. Young, Sergey V. Shmelkov, Michael F. Grunebaum, Timothy Cardozo

**Affiliations:** 1 Amherst College, Amherst, MA USA; 2 Department of Biochemistry and Molecular Pharmacology, NYU School of Medicine, New York, NY USA; 3 GeneCentrix Inc., New York, NY USA; 4 Department of Pathology, NYU School of Medicine, New York, NY USA; 5 Department of Neuroscience and Physiology, NYU School of Medicine, New York, NY USA; 6 Department of Psychiatry, NYU School of Medicine, New York, NY USA; 7 Department of Psychiatry, Columbia University Medical Center, New York, NY USA; 8 New York State Psychiatric Institute, New York, NY USA

**Keywords:** Bupropion, Nervous system tissues, Antidepressant

## Abstract

Despite decades of clinical use and research, the mechanism of action (MOA) of antidepressant medications remains poorly understood. Selective serotonin reuptake inhibitors (SSRIs) and serotonin-norepinephrine reuptake inhibitors (SNRIs) are the most commonly prescribed antidepressants—atypical antidepressants such as bupropion have also proven effective, while exhibiting a divergent clinical phenotype. The difference in phenotypic profiles presumably lies in the differences among the MOAs of SSRIs/SNRIs and bupropion. We integrated the ensemble of bupropion’s affinities for all its receptors with the expression levels of those targets in nervous system tissues. This “combined target tissue” profile of bupropion was compared to those of duloxetine, fluoxetine, and venlafaxine to isolate the unique target tissue effects of bupropion. Our results suggest that the three monoamines—serotonin, norepinephrine, and dopamine—all contribute to the common antidepressant effects of SSRIs, SNRIs, and bupropion. At the same time, bupropion is unique in its action on 5-HT3AR in the dorsal root ganglion and nicotinic acetylcholine receptors in the pineal gland. These unique tissue-specific activities may explain unique therapeutic effects of bupropion, such as pain management and smoking cessation, and, given melatonin’s association with nicotinic acetylcholine receptors and depression, highlight the underappreciated role of the melatonergic system in bupropion’s MOA.

**Electronic supplementary material:**

The online version of this article (10.1007/s00213-018-4958-9) contains supplementary material, which is available to authorized users.

## Introduction

Depression is the world’s leading cause of disability (World Health Organization 2017). The prevalence of depression increased significantly in the USA from 2005 to 2015, especially among youth (Weinberger et al. 2017). Yet, the pathophysiology of depression and, in parallel, the mechanism of action (MOA) of antidepressants are poorly understood. Depression is a chronic mood disorder characterized by persistent feelings of sadness, guilt, and hopelessness (U.S. Department of Health and Human Services 2015). It can affect everyday life by significantly diminishing concentration, motivation, interest in hobbies, quality of sleep, and appetite while increasing the risk for suicide. Antidepressants are among the most commonly prescribed medications in the USA and a mainstay of treatment for major depressive disorder (MDD) (National Center for Health Statistics 2017), but their mechanisms of action (MOAs) are still imprecisely known. The dominant theory is that key neurotransmitters such as serotonin (5-HT) and norepinephrine (NE) have diminished activity in depression, so most antidepressants work to enhance their neurotransmission (Elena et al. 2015). The most commonly prescribed antidepressants are selective serotonin reuptake inhibitors (SSRIs) and serotonin-norepinephrine reuptake inhibitors (SNRIs) (Preskorn et al. 2004). SSRIs such as fluoxetine increase 5-HT neurotransmission by blocking 5-HT transporters, and SNRIs such as duloxetine and venlafaxine increase both 5-HT and NE neurotransmission by blocking both of their transporters (Elena et al. 2015).

Bupropion (BP; Wellbutrin) is an atypical antidepressant in several respects. First, in contrast to SSRIs and SNRIs, BP’s theorized MOA is norepinephrine-dopamine reuptake inhibition (NDRI) (Stahl et al. 2004). BP’s primary side effects of dry mouth, activation, and insomnia are also distinct, as are its benefits of the absence of more common serotonergic antidepressant side effects such as sexual dysfunction, weight gain, and sedation (Stahl et al. 2004). BP has been shown to increase the neurotransmission of both dopamine (DA) and NE by blocking their respective transporters, thereby increasing their concentration in the synaptic cleft and activating their respective receptors (Stahl et al. 2004). However, BP’s clinical efficacy is likely not due solely to its inhibition of DA and NE reuptake. Not only is its inhibition of either the DA transporter or the NE transporter modest at best (Meyer et al. 2002; Learned-Coughlin et al. 2003; Tatsumi et al. 1997), but there is also no correlation between its clinical efficacy and DA transporter occupancy (Argyelán et al. 2005). It has also been shown to influence 5-HT neurotransmission, the primary target of more typical antidepressants, without inhibiting its reuptake (Ghanbari et al. 2011; Piacentini et al. 2003; Pandhare et al. 2017; Mansari et al. 2015). Due to its unique phenotypic effects, BP has been repurposed to facilitate smoking cessation, and although its efficacy in this area was initially believed to be due to its role as an NDRI (Stahl et al. 2004; Johnston et al. 2002), increasing evidence suggests that it is due instead to its non-competitive inhibition of several nicotinic acetylcholine receptors (nAChRs; also frequently abbreviated as “nACHRs”) (Crooks et al. 2014; Slemmer et al. 2000; Arias 2009; García-Colunga et al. 2011; Vásquez-Gómez et al. 2014; Miller et al. 2002). Ultimately, the absence of a clear MOA precludes the development of other bupropion-like compounds, or even improvement upon the existing scaffold.

Previously, we developed a novel in silico method for inferring, in vivo, the molecular target engagement signature of the MOA of drugs and applied it to a case study of the atypical antipsychotic drug clozapine (Shmelkov et al. 2015; Cardozo et al. 2017). This “historeceptomic” (HR) method integrates the affinities between a given drug and its complete ensemble of protein targets with the transcriptional levels of those targets in tissues of the body. By doing so, it contextualizes the drug’s molecular target engagement in broader physiologic terms, thereby potentially unveiling previously unsuspected MOAs for both therapeutic and side effects. The clozapine case study reduced uncertainty in the data by a differential approach: subtracting the HR profile of the typical antipsychotic drug chlorpromazine from that of clozapine to determine the mechanism underpinning the latter’s atypicality (atypia). This paper presents an analogous case study to isolate the atypical MOA of BP, using the more typical antidepressants duloxetine, fluoxetine, and venlafaxine as comparators. It is worth noting that duloxetine was included in the study as a representative SNRI because it has a more prominent effect on the NE system than venlafaxine does: duloxetine has only 10× more affinity for the 5-HT transporter than for the NE transporter, whereas venlafaxine has 30× more affinity for the 5-HT transporter (Stahl et al. 2005). However, venlafaxine was also included not only because it is the oldest and most commonly used SNRI but also because duloxetine is repurposed to treat other conditions such as fibromyalgia, which may complicate its HR profile (Lilly and Cymbalta 2017).

## Methods

The methods used in this study were previously described in a case study consisting of historeceptomic analysis of several schizophrenia drugs (Cardozo et al. 2017).

### Drug bioactivity data

The data on in vitro binding affinities (*K*_i_) of the drugs to various protein targets were downloaded from ChEMBL (https://www.ebi.ac.uk/chembl/, accessed on 31 July 2017) (Gaulton et al. 2012). It was filtered according to the following protocol.

1. All records with the “STANDARD_TYPE” other than “*K*_i_” were excluded;
2. All records with the “RELATION” other than “=”were excluded;
3. All records with the “STANDARD_UNITS” other than “nM” or “μM” were excluded;
4. All records with the “TARGET_TYPE” other than “single protein” were excluded;
5. All records with “ACTIVITY_COMMENTS” equal to “inactive” or “inconclusive” were excluded.

Drug-target interactions with a recorded IC_50_ value but no *K*_i_ value were assigned an affinity value of 10 nM, since IC_50_ values are less precise measures of affinity. The only drug-target interaction this applies to is fluoxetine with cytochrome P450 2C19. Further drug-target interaction data were downloaded from DrugBank (https://www.drugbank.ca/, accessed on 31 July 2017) (Law et al. 2014). Because DrugBank does not include any affinity values with its drug-target interactions, they were assigned an affinity value of 1.00 nM. These value assignments, while somewhat arbitrary, were chosen based on the distribution of all *K*_i_ values vs. IC_50_ values in ChEMBL. Ten nanomolars was chosen as the midpoint of the *K*_i_/*K*_d_ distribution for compounds that are not approved drugs with known targets, while 1 nM was chosen as that of approved drugs with known targets. The only drug-target interaction this applies to is BP with the nAChR α3 subunit.

A recent study found that BP binds to 5-HT3AR, so its published *K*_i_ value was also included in the data (Pandhare et al. 2017). Within the filtered data set, only the smallest (strongest) affinity value was used for each target; if no human data were available, the smallest affinity from other mammals was used instead.

### Tissue-specific gene expression data

Data for tissue expression levels of the genes that encode the various protein targets (tissues listed in Table S1) were downloaded from BioGPS (http://biogps.org/, accessed on 6 July 2017) (Wu et al. 2009; Wu et al. 2013). Importantly, the data for both human tissue and rat tissue were used to produce separate HR profiles for each drug, since the human data lacked important brain structures (e.g. the hippocampus, ventral tegmental area, dorsal raphe, locus coeruleus, and nucleus accumbens) and almost all BP targets are nearly identical in sequence between rat and human. The human data were downloaded from the data set “GeneAtlas U133A, gcrma,” while the rat data were downloaded from the data set “GeneAtlas RGU34A, gcrma.” If multiple strains of rats or multiple probes for either species were included for a given gene, the median of the expression values was used. Since the current study is interested in non-diseased human tissues, data for cell lines and diseased (cancer) tissues were excluded.

Importantly, the rat data were downloaded for only the genes that had statistically significant drug-target interactions within the human data. Unfortunately, the following human target outliers were not found in the rat data, and their respective genes had to be excluded from the analysis of the rat data: cytochrome P450 2C8, receptor-interacting serine/threonine-protein kinase 1, prostaglandin reductase 2, E3 ubiquitin-protein ligase UHRF1, and histamine H3 receptor.

### Target tissue scores

The target tissue scores (Tables S2–9) were calculated as the product of each drug-target affinity value on a logarithmic scale and the normalized expression level of the given target’s gene in each tissue (i.e., the *z*-score):

$$ \mathrm{Score}=-{\log}_{10}\ \mathrm{Affinity}\times z $$

We hypothesized that high scores imply a more significant phenotypic contribution by that target tissue pair.

### Statistical analysis

The distribution of all target tissue scores was assumed to be nearly normal with outliers, which represent the important tissue-specific drug-target interactions, i.e., the drug’s HR profile.

The rest of the scores would represent background noise, i.e., interactions of unknown or no physiological significance. A generalized extreme Studentized deviate test was used to detect the outliers (*α* = 0.001) (Rosner 1983). In order to improve the specificity of the method, only interactions with tissues of neural origin were included in the analysis. Although doing so may exclude interactions involved in the drug’s side effects, it allows for a clearer profile of the main MOA.

## Results

We generated the distribution of target tissue scores for each drug—BP, duloxetine, fluoxetine, and venlafaxine—and found the statistically significant outliers (see the “Methods” section) for the most complete data set we could assemble approximating human physiology (which includes both human and rat data, see the “Methods” section; Table 1). Only target tissue pairs with sufficiently high affinities of the target to BP and sufficiently high expression of the target in a specific tissue are identified by this method, which means that (1) previously accepted receptors for these drugs (such as the NE transporter for BP) may be found to be less significant than previously thought while unexpected receptors may emerge, and (2) the tissue locations of significant actions of the drug are precisely mapped. Experimentally determined affinities from previous studies for each drug and its respective targets are represented in the dataset, and the outliers include only nervous system tissue (see the “Methods” section). The full set of outliers represents the HR profile: the target tissue interactions most important for each drug’s MOA.

**Table 1:**
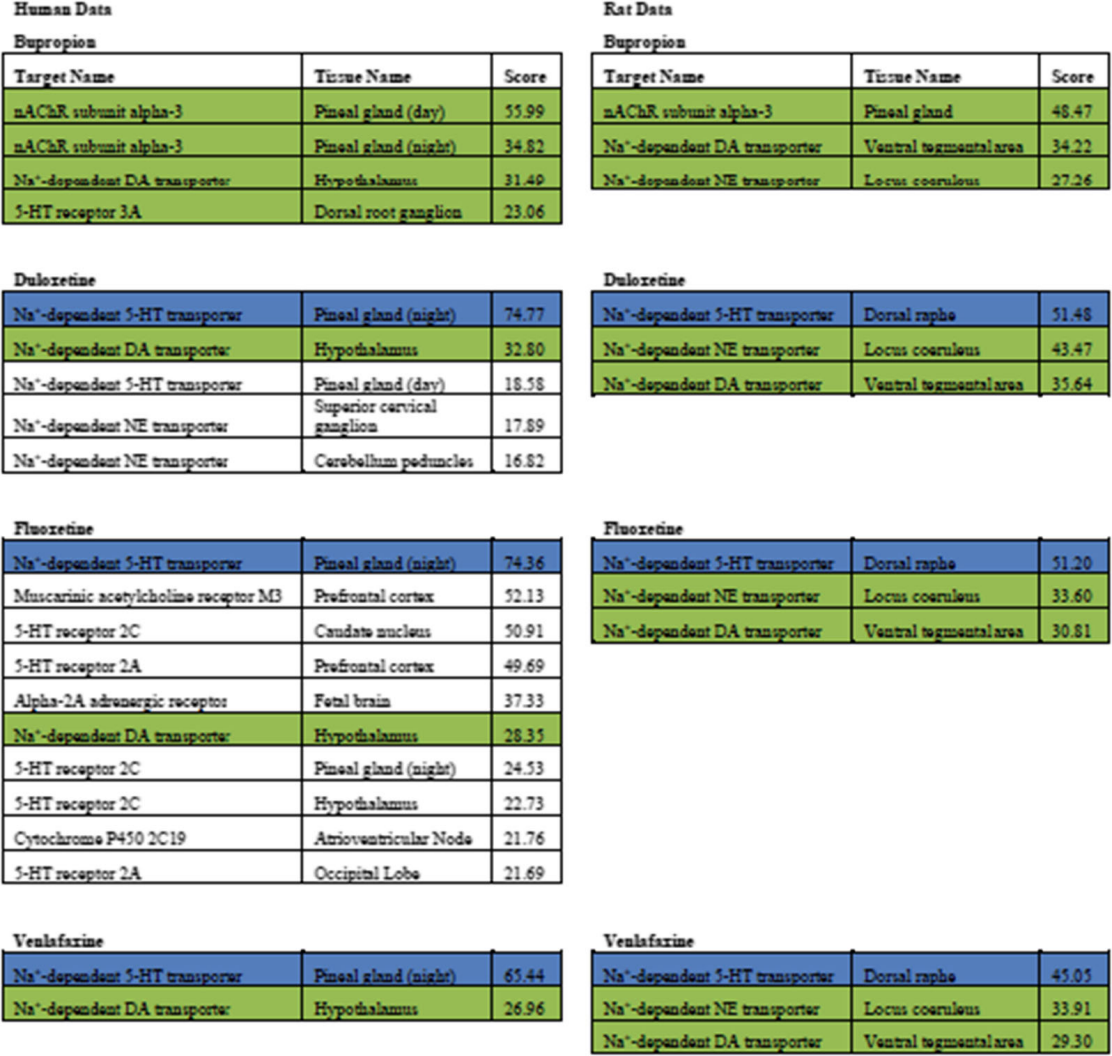
Statistically significant target tissue interactions for human data

The experimental binding affinities of BP, duloxetine, fluoxetine, and venlafaxine to various protein targets were integrated with each target’s gene expression data for an array of human tissues and rat tissues to generate a historeceptomic score. For each drug, the scores’ distribution was assumed to be nearly normal, with the outliers listed above (extreme Studentized deviate test, *α* = 0.001). The cells in green are the target tissue interactions that are part of BP’s outliers, while the cells in blue are the target tissue outliers that are shared only by the other three drugs

The common components for all four drugs are the DA transporter in the hypothalamus (human data) and ventral tegmental area (rat data), and the NE transporter in the locus coeruleus (rat data) (Table 1). Interestingly, the NE transporter did not appear as an outlier for either BP or venlafaxine for the human data, despite the fact that both drugs are known to inhibit NE reuptake. On the other hand, the three negative controls for BP’s atypia differ from BP in that they target the 5-HT transporter in the pineal gland (night) (human data) and in the dorsal raphe (rat data) (Table 1). BP’s unique outliers are the 5-HT3AR in the dorsal root ganglion (human data) and the nAChR α3 subunit in the pineal gland (both night and day) in both the human and the rat data (Table 1).

## Discussion

The HR profiles of the four drugs indeed reinforce that their MOA commonality lies in their shared action on all three monoaminergic systems. DA and NE in particular are prominent in the same tissues for all four drugs. The fact that the three typical antidepressants target the DA and NE transporters in the same specific tissues is particularly interesting because none of them are known to inhibit DA reuptake, while fluoxetine is thought to inhibit solely 5-HT reuptake.

Furthermore, the fact that BP targets both DA and NE transporters is consistent with previous findings that it requires functioning DA neurons and NE neurons for proper clinical efficacy (Cooper et al. 1980; Cryan et al. 2001).

Importantly, BP’s action on the 5-HT system differs from the other three drugs in that it does not inhibit 5-HT reuptake (Ghanbari et al. 2011; Piacentini et al. 2003; Pandhare et al. 2017; Mansari et al. 2015), and its most prominent 5-HT protein target is expressed outside of the brain, in the dorsal root ganglion. Although these results suggest that all three monoaminergic systems contribute to all four drugs’ antidepressant effects, BP’s action on the 5-HT system may be more associated with a sensory side effect than its main MOA. Research on 5-HT3 receptors remains somewhat ambiguous. Several antidepressants, including fluoxetine, have been found to inhibit the receptor (Eisensamer et al. 2003), which is also expressed in the hippocampus and the amygdala (Tecott et al. 1993). While 5-HT3 receptor agonists may diminish antidepressants’ effects (Nakagawa et al. 1998), the action of antagonists on anxiety remains unclear (Rodgers et al. 1995). At the same time, these antagonists are known to modulate pain perception and are used to prevent opioid dependence (Liang et al. 2011; Roychoudhury and Kulkarni 1996). Indeed, BP has been found to inhibit the 5-HT3AR (Pandhare et al. 2017) and reduce neuropathic pain (Hoshino et al. 2015). Although this effect has been connected to the DA and NE systems, our findings suggest the new hypothesis that it may in fact be related to BP’s antagonism against the receptor in the dorsal root ganglion.

Aside from the 5-HT3AR, BP’s only unique outlier is the nAChR in the pineal gland. This interaction was present in DrugBank as a consensus literature interaction and was thus assigned a virtual affinity value, so it may be a false positive. However, although DrugBank listed only the α3 subunit as its nAChR protein target, BP has been shown to bind to several nAChRs, including α3β2, α3β4, α4β2, α4β4, and α7 (Crooks et al. 2014; Slemmer et al. 2000; Arias 2009; García-Colunga et al. 2011; Vásquez-Gómez et al. 2014; Miller et al. 2002). This finding may be consistent with growing evidence that nAChR inhibition may produce antidepressant effects (Arias 2009; García-Colunga et al. 2011; Philip et al. 2010). One explanation for the connection between nAChR inhibition and depression is the cholinergic-adrenergic theory of depression: hyperactivation of the cholinergic system over the adrenergic system may contribute to depression, which should therefore be alleviated by nAChR inhibition (Shytle et al. 2002). However, this theory does not provide a specific biochemical pathway that underlies this phenomenon. Another possible explanation is nAChR’s interaction with the DA system: non-α7 nAChRs at the preterminal, somatic, and dendritic regions of GABAergic neurons inhibit DA neurons in the VTA upon activation (Arias 2009), so the receptor’s inhibition should improve DA neurotransmission.

However, this fails to account for the fact that activation of non-α7 nAChRs on DA neurons and presynaptic α7 nAChRs on glutamatergic neurons increase DA excitability. On the other hand, α7-nAChR inhibition may exert antidepressant effects via activation of the mTOR pathway and upregulation of synaptic proteins, ameliorating stress/depression-induced atrophy in the hippocampus and PFC (Singh et al. 2013). In addition, α7-nAChR-mediated d-serine reduction may indirectly decrease NMDA receptors activation, thus being associated with antidepressant effects (Singh et al. 2016).

However, although these proposed mechanisms are intriguing and should be investigated further, they ignore the fact that nAChR’s only significant target tissue pair was found to be with the pineal gland.

**Fig. 1:**
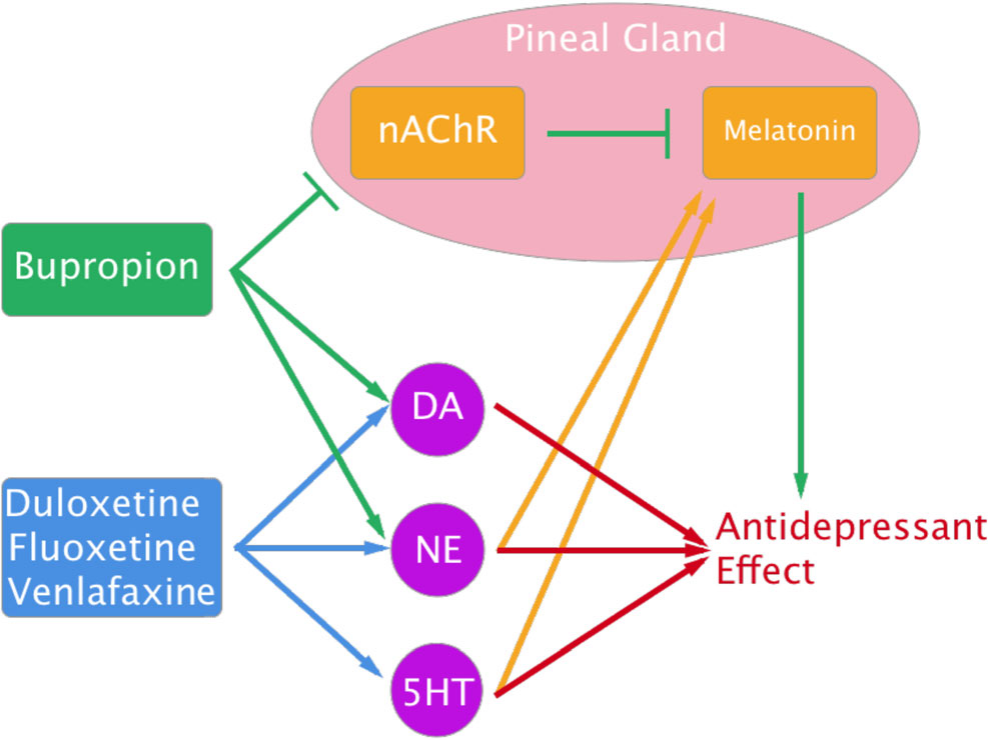
The antidepressant melatonergic pathway. Duloxetine, fluoxetine, and venlafaxine were all found to increase DA, NE, and 5-HT neurotransmission by inhibiting their reuptake. BP was found to increase only DA and NE neurotransmission, but it was also found to inhibit nAChRs in the pineal gland. nAChR activation has been shown to inhibit MT synthesis, which is diminished in depression and can elicit antidepressant effects when enhanced. The proposed melatonergic role in depression is consistent with the fact that 5-HT is in the biosynthetic pathway of MT and that NE release from superior cervical ganglion neurons stimulates MT synthesis and release

In light of BP’s activity specifically in the pineal gland, the melatonergic (also frequently termed “melatoninergic”) pathway may have an underestimated role in its MOA (Fig. 1). nAChR activation in the pineal gland inhibits melatonin (MT) synthesis (Yamada et al. 1998), and depression is associated with diminished levels of nocturnal MT secretion (Malhotra et al. 2004; Beck-Friis et al. 1985; Brown et al. 1985). Indeed, MT receptor agonists have been shown to effectively treat depression (Olié and Kasper 2007), though the precise biochemical mechanisms by which MT influences depression has yet to be fully elucidated. Thus, in addition to facilitating smoking cessation, BP’s inhibition of nAChRs may contribute significantly to its antidepressant effects via melatonergic activity. In fact, this theory is consistent with the fact that the 5-HT transporter in the pineal gland at night is an outlier for the other three drugs for the human data: 5-HT is an intermediate in MT’s biosynthetic pathway (Miles and Philbrick 1988), so these other drugs may also contribute to MT synthesis. This is also consistent with the fact that one of duloxetine’s outliers for the human data is the NE transporter in the superior cervical ganglion, since NE release from superior cervical ganglion neurons stimulates MT synthesis and release (Cardinali and Vacas 1987). Indeed, these parallels may contribute to the sedative effects some patients experience while using SSRIs and SNRIs (Anderson et al. 2012). While these speculations are largely theoretical, as the literature is currently lacking in studies that directly measure BP’s effects on the pineal gland and MT synthesis, these findings show promise for potential future investigations.

BP’s connection to the pineal gland may also contribute to its antinicotinic effects. The medial habenula is a group of nerve cells near the pineal gland that sends signals to both the pineal gland and the interpeduncular nucleus (which thus forms the habenulo-interpeduncular pathway). It has been found that these neurons contain nAChRs and are sensitive to nicotine (Shih et al. 2014).

This may be evidence that BP’s action on nAChRs specifically in or near the pineal gland contributes to its therapeutic effects against nicotine addiction. Given the further connection found between this pathway’s cholinergic signaling and depression (Han et al. 2017), it seems worthwhile to consider for further study.

It is worth addressing the fact that BP—along with duloxetine, fluoxetine, and venlafaxine—has been shown to increase rates of insomnia (Alberti et al. 2015). MT deficiency is associated with insomnia and other sleep disorders, which are thus treated with MT administration (Yamada et al. 1998). BP’s affinity for nAChRs (Crooks et al. 2014; Slemmer et al. 2000; Arias 2009; García-Colunga et al. 2011; Vásquez-Gómez et al. 2014; Miller et al. 2002) and the corresponding high expression of nAChRs in the pineal gland that results in a significant HR score suggests a direct influence of BP on MT synthesis or release. Here, a seeming paradox arises: BP promotes MT synthesis but still antagonizes sleep; indeed, studies have found conflicting results regarding the relationship between sleep and depression (Roberts and Duong 2014; Dopierała and Rybakowski 2015). Fortunately, this paradox can be resolved upon consideration of the complex and multifaceted interactions BP and the other antidepressants have with the brain. Importantly, DA and NE stimulate arousal (Jones 2005a; Jones 2005b; Osaka and Matsumura 1995), which is consistent with the fact that all four drugs increase both monoamines’ neurotransmissions and would in this way increase rates of insomnia. Furthermore, although acetylcholine has been shown to inhibit MT synthesis, it also promotes rapid eye movement (REM) sleep (Jones 2005a; Jones 2004; Gillin and Sitaram 1984). Finally, BP’s amphetamine-like properties may outweigh the potential sedative melatonergic effect (Brensilver et al. 2013). An unclear but important relationship thus appears to exist among BP, MT, nAChRs, sleep, and depression, requiring further investigation.

The current pioneering version of historeceptomics is highly specific but not very sensitive. Thus, many target tissue pairs that are influential in the MOAs of the four drugs studied here may have barely reached statistical significance. Indeed, the reason that neither BP nor venlafaxine has the NE transporter as an outlier for the human data is likely that BP and venlafaxine both have relatively low affinity for the NE transporter, compared to their affinities for their other targets (Tatsumi et al. 1997; Stahl et al. 2005). Additionally, the human data do not include nucleus accumbens tissue, which has particularly high NE transporter expression levels as seen in the rat data. Thus, the scores of BP and venlafaxine for the NE transporter were too low in the human data for the outlier detection model to identify them, but this does not mean these interactions are not physiologically important.

In summary, historeceptomics (HR) is unique, but not yet perfect, in taking both molecular target engagement and the tissue-specific expression of drug targets into account to generate signatures for drug MOAs. Comparison of the HR profiles of the atypical antidepressant bupropion (BP) and three controls, duloxetine, fluoxetine, and venlafaxine, reveals potentially important information both about all four drug’s common antidepressant effects in vivo and about the tissue and molecular basis for BP’s atypia. Our findings are consistent with the growing appreciation of the role of melatonin and the pineal gland in both depression and the action of antidepressants. Future studies into the MOA of BP, and potentially of other antidepressants, should further explore their effects on the pineal gland and specifically the melatonin system. Meanwhile, the MOA of antidepressants in general might be better viewed through the more complex prism of neurotransmission of all three monoamines: 5-HT, DA, and NE.

## Electronic supplementary material

ESM 1 (PDF 352 kb)
