# Supplementary material for: Molecular basis of atypicality of bupropion inferred from its receptor engagement in nervous system tissues
Source: Psychopharmacology (Berl). 2018 Jul 1;235(9):2643–50. doi: 10.1007/s00213-018-4958-9 (PMC6132670; doi:10.1007/s00213-018-4958-9)
Supplement: Supplementary file 1 — (PDF 352 kb) [file 213_2018_4958_MOESM1_ESM.pdf]

Supplemental Table 1. Tissues analyzed for protein target transcription levels

Human Nervous Tissue	Rat	Human Non-Nervous Tissue	Rat Non-Nervous Tissue
Amygdala	Amygdala	Adipocyte	Bone marrow
Atrioventricular node	Amygdala (central nucleus)	Adrenal cortex	Cornea
Caudate nucleus	Cerebellum	Adrenal gland	Endothelial cells
Cerebellum	Cerebral cortex	Appendix	Heart
Cerebellum peduncles	Dorsal raphe	BDCA4+ dentritic cells	Kidney
Ciliary ganglion	Dorsal root ganglion	Bone marrow	Large intestine
Cingulate cortex	Dorsal striatum	Bronchial epithelial cells	Skeletal muscle
Dorsal root ganglion	Frontal cortex	Cardiac myocytes	Small intestine
Fetal brain	Hippocampus	CD105+ endothelial cells	Spleen
Globus pallidus	Hypothalamus	CD14+ monocytes	Thymus
Hypothalamus	Locus coeruleus	CD19+ B cells (neg._sel.)	
Medulla oblongata	Nucleus accumbens (core)	CD33+ myeloid	
Occipital lobe	Nucleus accumbens (shell)	CD34+	
Olfactory bulb	Nucleus accumbens (whole)	CD4+ T cells	
Parietal lobe	Pineal gland	CD56+ NK cells	
Pineal gland (day)	Pituitary gland	CD71+ early erythroid	
Pineal gland (night)	Prefrontal cortex	CD8+ T cells	
Pituitary gland	Primary cortical neurons	Colon	
Pons	Ventral striatum	Fetal liver	
Prefrontal cortex	Ventral tegmental area	Fetal lung	
Spinal cord		Fetal thyroid	
Subthalamic nucleus		Heart	
Superior cervical ganglion		Kidney	
Temporal lobe		Liver	
Thalamus		Lung	
Trigeminal ganglion		Lymph node	
Whole brain		Ovary	
		Pancreas	
		Pancreatic islet	
		Placenta	
		Prostate	
		Retina	
		Salivary gland	
		Skeletal muscle	
		Skin	
		Small intestine	
		Smooth muscle	
		Testis	
		Testis germ cell	

		Testis intersitial	
		Testis leydig cell	
		Testis seminiferous tubule	
		Thymus	
		Thyroid	
		Tongue	
		Tonsil	
		Trachea	
		Uterus	
		Uterus corpus	
		Whole blood	

The gene expression levels of the drugs' protein targets were downloaded from BioGPS for the human data and the rat data. While the z-scores were calculated using all the tissues given, only the nervous system tissues were included in the statistical analysis. Importantly, the rat data was included in the study to supplement the human data's lack of key nervous system tissues.

Supplemental Table 2. Bupropion's HR scores for human data

	Sodium-dependent dopamine transporter	Sodium-dependent noradrenaline transporter	Aldo-keto reductase family 1 member C3	Neuronal acetylcholine receptor subunit alpha-3	5-HT receptor 3A
**CardiacMyocytes**	6.122656541	8.529743982	-1.091975723	-2.246548478	3.398476762
**Fetallung**	-6.12492882	-7.15453264	-1.679063608	-2.48116245	-3.335151108
**CerebellumPeduncles**	6.997484067	10.54629383	-1.675514105	-1.073478621	0.844342053
**pineal_day**	6.560070304	4.138146528	-1.645130355	55.99134251	2.314904461
**Testis**	-5.075135789	-1.687441932	-1.696243206	-2.192922428	-2.700487332
**FetalThyroid**	0.436277623	-1.77706637	-1.644278474	-1.743804254	-1.090608485
**BDCA4+_DentriticCells**	2.185932675	-1.328944181	-1.489520119	-1.743804254	5.023835214
**retina**	5.466535897	4.04852209	-1.497329027	10.6739781	2.23750644
**TemporalLobe**	-3.937860006	-2.897371843	-1.69113192	-2.447646168	-2.096782764
**Fetalbrain**	-1.750791191	-1.553005275	-1.689002218	-1.576222845	-1.322802549
**TestisIntersitial**	-6.562342583	-0.656760897	-1.698940828	-2.28006476	-3.41254913
**SmoothMuscle**	3.498173964	2.256033333	4.404075559	-1.643255409	2.160108418
**CD4+_Tcells**	1.748518912	-0.656760897	-1.679063608	-1.408641437	-0.007036184
**OccipitalLobe**	-3.937860006	-3.569555127	-1.694681424	-2.213032197	-2.483772872
**Prostate**	4.373001489	2.256033333	-1.674804204	-0.503701833	1.153934139
**Lymphnode**	-6.12492882	-5.810166073	-1.698230927	-2.347097323	-2.793364958
**CD19+_BCells(neg._sel.)**	0.873691386	0.687605671	-1.684742814	-1.978418225	-0.084434205
**Hypothalamus**	31.49265479	0.911666765	-1.679063608	-1.207543747	-0.007036184
**UterusCorpus**	-2.188204954	-0.208638708	-1.693261623	-2.145999633	-1.013210463
**Heart**	6.560070304	9.201927266	-1.608073538	-0.403152988	3.708068848
**Lung**	2.185932675	1.583850049	1.270573834	-2.916874111	0.302555902
**Salivarygland**	-6.562342583	-6.482349356	-1.699650729	-2.313581042	1.076536117
**Uterus**	-4.59398065	-2.561280201	-1.632920062	-2.296822901	-2.290277818
**Skin**	-8.749411398	-2.673310748	-1.698230927	-2.28006476	-3.102957044
**ParietalLobe**	0.873691386	1.583850049	-1.68048341	-2.347097323	-0.239230248
**Thalamus**	0.436277623	-0.656760897	-1.683323013	-2.246548478	-0.703618377
**Adipocyte**	3.498173964	-1.77706637	21.22801244	-1.542706564	-1.168006506
**PrefrontalCortex**	6.560070304	3.824460995	-1.665575495	-0.637766959	2.082710397
**AdrenalCortex**	2.623346438	3.600399901	-1.681903211	-1.643255409	0.766944031
**BronchialEpithelialCells**	-1.750791191	-2.673310748	10.43255237	-2.414129887	-1.322802549
**AtrioventricularNode**	-5.687515057	-1.104883086	-1.696811126	-2.347097323	-2.174180786
**Liver**	8.747139118	10.99441602	0.977384841	0.635851743	3.475874784
**Placenta**	0.436277623	9.201927266	-1.687582417	-2.145999633	-0.935812442
**Amygdala**	0.436277623	-0.208638708	-1.684032913	-2.045450788	-0.471424313
**CD33+_Myeloid**	8.309725355	6.289133036	-1.667705197	-1.542706564	2.77929259
**PancreaticIslet**	1.311105149	0.463544576	-0.565939298	-1.576222845	-0.161832227

**TestisLeydigCell**	-1.750791191	0.463544576	-1.684742814	-1.67677169	-1.168006506
**CingulateCortex**	0.436277623	-0.208638708	-1.684032913	-1.542706564	-0.239230248
**CD34+**	8.309725355	5.616949752	3.929151985	-1.810836817	2.23750644
**Trachea**	-6.12492882	-5.586104978	-0.721407553	-0.939413494	-3.257753087
**SuperiorCervicalGanglion**	2.623346438	11.21847712	-1.666285396	-0.23557158	14.23419977
**Pancreas**	-5.250101294	-5.137982789	-0.02641476	-2.548195013	-3.722141216
**Bonemarrow**	-2.188204954	2.704155522	-1.646408176	-1.375125156	-0.31662827
**CD14+_Monocytes**	1.311105149	0.463544576	-1.681193311	-3.017422956	-0.394026291
**MedullaOblongata**	-3.937860006	-3.569555127	-1.694681424	-2.011934507	-1.477598592
**Caudatenucleus**	-4.375273769	-3.793616221	-1.693261623	-2.07896707	-2.483772872
**SkeletalMuscle**	17.49541438	18.83655433	-1.663445793	-0.939413494	2.314904461
**Tongue**	-1.750791191	2.031972238	-1.666285396	-1.87786938	-0.781016399
**SubthalamicNucleus**	-2.188204954	0.911666765	-1.692551722	-2.045450788	-2.096782764
**Thyroid**	3.716880845	0.911666765	-1.655991835	-2.011934507	0.496050956
**Thymus**	-4.812687531	-6.482349356	-0.589366021	38.27463603	-3.876937259
**Cerebellum**	-0.219843021	2.031972238	-1.688292318	-0.436669269	-1.400200571
**OlfactoryBulb**	-7.437170109	-3.569555127	-1.703200232	-2.548195013	-3.799539237
**Fetalliver**	-6.12492882	-3.793616221	-1.281519212	-2.68226014	-2.793364958
**Adrenalgland**	-6.562342583	-4.465799505	-1.543472573	-2.112483352	-3.180355065
**WholeBlood**	1.748518912	2.031972238	-1.663445793	-2.246548478	0.147759859
**small_intestine**	-2.188204954	-14.99667095	16.97002799	-4.257525377	-5.037907581
**TestisGermCell**	-5.687515057	-5.586104978	-1.701780431	-2.514678731	-3.41254913
**TrigeminalGanglion**	4.810415252	6.51319413	-1.685452715	-1.542706564	1.695720289
**colon**	-1.313377428	-1.328944181	4.158449914	-1.576222845	-1.090608485
**TestisSeminiferousTubule**	-5.687515057	-4.913921694	-1.698230927	-2.28006476	-2.948161001
**CD105+_Endothelial**	-0.875963665	-1.328944181	26.79221419	-2.514678731	-1.013210463
**Tonsil**	-2.188204954	-1.77706637	-1.687582417	-1.67677169	2.469700504
**CiliaryGanglion**	9.184552881	-4.465799505	-1.701780431	-2.782808985	8.274552117
**GlobusPallidus**	-9.624238923	-6.258288262	-1.706039835	-2.916874111	-4.573519452
**CD8+_Tcells**	-2.188204954	-3.569555127	-1.692551722	-3.151488083	-1.787190678
**pineal_night**	6.035173788	3.690024338	-1.666995296	34.82245902	1.757638706
**Appendix**	-0.438549903	4.272583184	-1.681903211	-1.475674	7.964960031
**Pituitary**	4.810415252	2.928216617	-1.674094303	-0.872380931	0.999138096
**Pons**	-1.313377428	-0.880821991	-1.689712119	-1.87786938	-1.554996614
**Spinalcord**	0.873691386	0.239483482	-1.679773509	-1.341608874	-0.161832227
**DorsalRootGanglion**	-2.188204954	-2.225188559	-1.70107053	-2.447646168	23.05757422
**Wholebrain**	-5.687515057	-5.586104978	-1.698230927	-2.615227576	-3.257753087
**Kidney**	-7.437170109	-5.137982789	2.030877492	-2.246548478	-3.41254913
**CD56+_NKCells**	5.247829015	3.824460995	11.45480939	-0.905897212	1.308730182

**Ovary**	-12.2487215	-6.034227167	-1.711719041	-2.615227576	-4.263927366
**CD71+_EarlyErythroid**	-3.500446243	-2.673310748	-0.671004603	-2.715776421	-1.8645887

Bupropion's HR profile of scores were calculated by combining target affinities and target-tissue expression levels according to the formula specified in the Methods. The left column lists all of the human tissues for which expression levels were measured, while the header lists bupropion's targets.

Supplemental Table 3. Duloxetine's HR scores for human data

	Sodium-dependent serotonin transporter	Sodium-dependent noradrenaline transporter	Sodium-dependent dopamine transporter	Cytochrome P450 2B6	Receptor-interacting serine/threonine-protein kinase 1	E3 ubiquitin-protein ligase UHRF1
**OlfactoryBulb**	-3.714021	-5.692736	-7.746364	-0.922233	-2.688727	-0.997838
**SuperiorCervicalGanglion**	6.411268	17.891256	2.732410	0.949631	0.071610	-0.999924
**WholeBlood**	-1.481674	3.240595	1.821212	-0.652415	4.447753	-0.996795
**Lung**	6.730174	2.525928	2.276811	-0.323574	-0.736294	-0.997838
**Fetallung**	-3.315387	-11.410067	-6.379568	-0.694574	-2.621402	-0.996795
**PrefrontalCortex**	-0.206047	6.099260	6.832800	-0.534369	0.273585	-1.005138
**Ovary**	-4.670741	-9.623401	-12.757952	-0.441619	-3.294655	-0.996795
**ParietalLobe**	-1.083040	2.525928	0.910014	-0.618687	-0.870944	-0.996795
**retina**	-0.564817	6.456593	5.693803	-0.475346	0.307248	-1.001488
**CD4+_Tcells**	-1.401947	-1.047404	1.821212	-0.686142	20.673147	-0.999924
**CD56+_NKCells**	-0.684407	6.099260	5.466003	-0.601824	23.568134	-0.998881
**Uterus**	-2.996481	-4.084736	-4.784972	-0.833699	-2.116462	-0.997838
**Appendix**	-1.800581	6.813927	-0.456782	0.494313	-1.005595	-0.996795
**TestisIntersitial**	-3.315387	-1.047404	-6.835167	-0.677710	-2.352101	-0.996795
**Thymus**	-3.793748	-10.338067	-5.012771	-0.905369	-2.688727	21.969546
**Adipocyte**	-2.199214	-2.834070	3.643608	-0.728301	-1.207571	-0.996795
**Kidney**	-3.395114	-8.194068	-7.746364	-0.821051	-2.419426	-0.995752
**Placenta**	9.680062	14.675257	0.454416	-0.728301	2.697296	-0.996795
**Amygdala**	-1.641127	-0.332738	0.454416	-0.711437	-1.005595	-0.999924
**Thalamus**	-1.960034	-1.047404	0.454416	-0.711437	-1.140246	-0.994709
**Pancreas**	-3.554568	-8.194068	-5.468370	-0.357301	-2.486751	-0.996795
**BronchialEpithelialCells**	-2.278941	-4.263403	-1.823579	-0.762028	-1.342221	-0.996795
**GlobusPallidus**	-4.112654	-9.980734	-10.024359	-0.896937	-2.958028	-0.996795
**AdrenalCortex**	-1.561400	5.741927	2.732410	-0.458483	-0.938270	-0.995752
**CerebellumPeduncles**	-1.003314	16.819257	7.288399	-0.542801	-0.265017	-0.995752
**CiliaryGanglion**	-4.272108	-7.122069	9.566393	-0.483778	-2.756052	-0.997838
**BDCA4+_DentriticCells**	-2.039761	-2.119404	2.276811	-0.694574	3.505199	-0.786123
**Spinalcord**	-1.481674	0.381929	0.910014	-0.669278	-0.736294	-0.995752
**FetalThyroid**	-2.278941	-2.834070	0.454416	-0.340437	-1.544197	-0.994709
**Lymphnode**	-3.235661	-9.266068	-6.379568	-0.880074	-1.409547	-0.996795
**DorsalRootGanglion**	-3.155934	-3.548736	-2.279178	-0.332006	-2.621402	-0.996795
**Skin**	-3.953201	-4.263403	-9.113161	-0.104346	-0.870944	-0.997838
**pineal_night**	74.768925	5.884860	6.286081	-0.556292	0.287051	-0.998881
**CD105+_Endothelial**	-2.199214	-2.119404	-0.912381	-0.736733	-1.140246	11.166887
**CingulateCortex**	-1.880307	-0.332738	0.454416	-0.643983	-1.072920	-0.996795

**SkeletalMuscle**	-0.524954	30.040585	18.222772	-0.205528	0.475561	-0.996795
**colon**	-2.039761	-2.119404	-1.367980	-0.711437	-1.140246	-0.818454
**small_intestine**	24.429498	-23.916730	-2.279178	-1.276369	-1.409547	-0.978022
**Adrenalgland**	-3.235661	-7.122069	-6.835167	-0.821051	-2.217450	-0.995752
**Caudatenucleus**	-1.083040	-6.050069	-4.557172	-0.804187	-2.015474	-0.994709
**Thyroid**	-1.322220	1.453929	3.871407	-0.344653	0.744862	-0.997316
**SubthalamicNucleus**	-2.597847	1.453929	-2.279178	-0.677710	-1.813498	-0.996795
**CD19+_BCells(neg._sel.)**	-1.641127	1.096595	0.910014	-0.686142	-0.601643	-0.998881
**CD14+_Monocytes**	-1.800581	0.739262	1.365613	-0.677710	-0.197692	-0.997838
**TrigeminalGanglion**	-1.322220	10.387259	5.010404	1.185722	-0.332342	-0.997838
**TestisGermCell**	-3.554568	-8.908735	-5.923969	-0.905369	-2.554077	-0.997838
**Prostate**	-0.923587	3.597928	4.554806	-0.593392	-0.265017	-0.996795
**OccipitalLobe**	-2.837027	-5.692736	-4.101573	-0.821051	-1.948149	-0.995752
**CD34+**	0.033133	8.957926	8.655195	-0.542801	0.744862	35.088551
**Pituitary**	-0.604680	4.669927	5.010404	-0.559665	-0.197692	-0.995752
**Cerebellum**	2.385070	3.240595	-0.228983	-0.707221	-1.375884	-0.996274
**CardiacMyocytes**	-0.206047	13.603258	6.377201	-0.391028	0.946838	-1.010353
**PancreaticIslet**	-1.401947	0.739262	1.365613	-0.669278	2.091368	-0.996795
**Liver**	1.388487	17.533923	9.110794	43.479718	1.081489	-0.997838
**TemporalLobe**	-2.837027	-4.620736	-4.101573	-0.762028	-1.880824	-0.996795
**CD33+_Myeloid**	0.033133	10.029926	8.655195	-0.525937	0.812188	-0.966550
**AtrioventricularNode**	-3.953201	-1.762070	-5.923969	-0.391028	-2.352101	-0.997838
**TestisSeminiferousTubule**	-2.837027	-7.836735	-5.923969	-0.559665	-2.284775	-0.997838
**pineal_day**	18.577559	6.599527	6.832800	-0.529310	0.367841	-0.998881
**Fetalliver**	0.750673	-6.050069	-6.379568	-0.821051	-2.015474	-0.991580
**TestisLeydigCell**	-2.199214	0.739262	-1.823579	-0.281415	-1.274896	-0.997838
**Bonemarrow**	-0.046594	4.312594	-2.279178	-0.660846	-1.005595	-0.720419
**Testis**	-2.996481	-2.691137	-5.286130	-0.539428	-2.150125	-0.997421
**SmoothMuscle**	-1.242494	3.597928	3.643608	-0.601824	0.071610	-0.996795
**CD71+_EarlyErythroid**	-2.518121	-4.263403	-3.645974	-0.770460	0.273585	3.880984
**UterusCorpus**	-2.358667	-0.332738	-2.279178	-0.753596	-1.880824	-0.998881
**Pons**	-1.322220	-1.404737	-1.367980	-0.534369	-1.544197	-0.996795
**MedullaOblongata**	-2.199214	-5.692736	-4.101573	-0.804187	-1.948149	-0.997838
**Heart**	0.352040	14.675257	6.832800	-0.340437	0.812188	-1.007224
**Fetalbrain**	-1.720854	-2.476737	-1.823579	-0.745165	-1.274896	-0.996795
**Salivarygland**	-3.634294	-10.338067	-6.835167	-0.601824	-2.554077	-0.996795
**Tongue**	-2.199214	3.240595	-1.823579	-0.272983	-1.409547	-0.995752
**Hypothalamus**	-1.641127	1.453929	32.801937	-0.694574	-0.870944	-0.997838
**Tonsil**	-2.278941	-2.834070	-2.279178	-0.728301	-1.342221	-0.993666

**CD8+_Tcells**	-2.757301	-5.692736	-2.279178	-0.787324	26.665097	-0.999924
**Wholebrain**	-3.155934	-8.908735	-5.923969	-0.905369	-2.486751	-0.998881
**Trachea**	-3.315387	-8.908735	-6.379568	-0.576528	-2.284775	-0.996795

Duloxetine’s HR profile of scores were calculated by combining target affinities and target-tissue expression levels according to the formula specified in the Methods. The left column lists all of the human tissues for which expression levels were measured, while the header lists duloxetine’s targets.

Supplemental Table 4a. Fluoxetine's HR scores for human data

	Sodium-dependent serotonin transporter	Alpha-2A adrenergic receptor	Sodium-dependent noradrenaline transporter	Sodium-dependent dopamine transporter	Histamine H3 receptor	Cytochrome P450 2C8	Alpha-2B adrenergic receptor	Muscarinic acetylcholine receptor M3
**Prostate**	-0.91852	1.16006	2.78092	3.93656	1.64970	-0.40389	6.37024	-0.67104
**Adipocyte**	-2.18715	15.28145	-2.19052	3.14904	-1.89712	-0.42753	-0.37281	-1.04361
**Thymus**	-3.77294	-8.06206	-7.99054	-4.33236	-6.55864	-0.45209	-8.56079	-1.41618
**PrefrontalCortex**	-0.20492	1.44826	4.71427	5.90534	2.96708	-0.39694	9.74177	52.13444
**Cerebellum**	2.37199	-2.29823	2.50473	-0.19790	3.52444	-0.41316	-1.09528	-1.08087
**colon**	-2.02857	-3.30690	-1.63814	-1.18230	-1.28909	-0.41826	0.59049	-0.67104
**AtrioventricularNode**	-3.93152	-5.18015	-1.36195	-5.11987	0.53498	-0.37237	-8.56079	-1.44102
**Heart**	0.35011	5.91522	11.34286	5.90534	5.50052	-0.27272	10.70506	-0.42266
**CD71+_EarlyErythroid**	-2.50431	-4.74786	-3.29529	-3.15109	-1.99845	-0.42336	-1.81775	-1.14297
**Fetalbrain**	-1.71142	37.32810	-1.91433	-1.57605	-2.60648	-0.42985	-0.37281	2.95530
**CiliaryGanglion**	-4.24868	-5.75653	-5.50482	8.26789	-6.25463	-0.40018	-10.96902	-1.54037
**Thyroid**	-1.31497	0.22344	1.12378	3.34592	1.95371	-0.40783	4.44366	-0.80765
**TestisGermCell**	-3.53507	-7.48568	-6.88578	-5.11987	-6.05195	-0.43819	-6.15256	-1.34167
**CD19+_BCells(neg._sel.)**	-1.63213	-2.87462	0.84758	0.78649	-0.98508	-0.41780	2.51707	-0.86975
**SkeletalMuscle**	-0.52207	1.01597	23.21909	15.74929	14.41822	-0.27133	6.85189	-0.82007
**Uterus**	-2.98005	2.45693	-3.15719	-4.13548	-4.58256	-0.43054	-5.43009	-1.27957
**Kidney**	-3.37649	-5.61244	-6.33339	-6.69491	-5.03858	-0.42243	-7.11585	-1.36651
**Appendix**	-1.79071	0.43959	5.26665	-0.39478	-0.98508	-0.36079	0.10884	-0.74556
**CD14+_Monocytes**	-1.79071	-1.72185	0.57139	1.18025	1.64970	-0.40296	2.99872	-0.91942
**CerebellumPeduncles**	-0.99781	1.01597	13.00001	6.29910	7.52727	-0.38211	5.40695	-0.77040
**BDCA4+_DentriticCells**	-2.02857	-3.59510	-1.63814	1.96777	-0.78240	-0.41965	4.44366	-0.84491
**Pituitary**	-0.60136	1.16006	3.60950	4.33031	1.54836	-0.38860	6.37024	-0.74556
**retina**	-0.56172	4.33017	4.99046	4.92095	2.81508	-0.39022	8.29683	1.37809
**Testis**	-2.98005	-4.08502	-2.08004	-4.56861	-4.55216	-0.42975	-4.61129	-1.25722
**Amygdala**	-1.63213	0.87187	-0.25718	0.39274	6.41256	-0.42614	2.03542	6.63131
**Fetallung**	-3.29720	-6.04472	-8.81912	-5.51363	-6.45730	-0.45812	-6.63421	-1.31683
**TestisLeydigCell**	-2.18715	-2.73052	0.57139	-1.57605	-1.59310	-0.40667	0.10884	-1.04361
**Salivarygland**	-3.61436	-6.33291	-7.99054	-5.90739	-4.63323	-0.42568	-7.11585	-1.41618
**WholeBlood**	-1.47355	-1.14547	2.50473	1.57401	0.23097	-0.40992	3.48036	-0.82007
**CD34+**	0.03295	6.77980	6.92380	7.48038	3.98046	-0.39972	10.70506	-0.49718
**Caudatenucleus**	-1.07710	-5.03605	-4.67624	-3.93860	13.50618	-0.43170	-3.74433	-1.21748
**CardiacMyocytes**	-0.20492	12.11134	10.51429	5.51159	8.54064	-0.28153	13.11329	-0.37299

**CD105+_Endothelial**	-2.18715	-3.73919	-1.63814	-0.78854	-1.39043	-0.42150	0.59049	-0.96910
**Tonsil**	-2.26644	6.20342	-2.19052	-1.96981	-2.20113	-0.41548	-0.85445	-1.06845
**SuperiorCervicalGanglion**	6.37611	7.93256	13.82858	2.36152	2.56173	-0.29451	6.85189	-0.77040
**CD56+_NKCells**	-0.68065	-1.43366	4.71427	4.72407	1.85237	-0.41038	7.33353	-0.64621
**Fetalliver**	0.74656	-5.03605	-4.67624	-5.51363	-3.72119	-0.43031	-5.18927	-1.26715
**Skin**	-3.93152	-3.73919	-3.29529	-7.87618	2.96708	-0.39277	-9.04244	-1.11813
**Ovary**	-4.64513	-8.35025	-7.43816	-11.02624	-8.18004	-0.45302	-13.37726	-1.61489
**Wholebrain**	-3.13863	10.67038	-6.88578	-5.11987	-2.80915	-0.44375	-6.15256	5.51361
**Pancreas**	-3.53507	-1.86595	-6.33339	-4.72612	-5.34259	-0.43448	-7.59750	-0.86975
**BronchialEpithelialCells**	-2.26644	-4.02738	-3.29529	-1.57605	-3.31584	-0.42799	-0.37281	-1.06845
**AdrenalCortex**	-1.55284	-1.57775	4.43807	2.36152	1.95371	-0.36218	1.55378	-0.94426
**MedullaOblongata**	-2.18715	-4.89196	-4.40005	-3.54484	-2.70782	-0.42521	-3.26268	-0.09977
**Thalamus**	-1.94928	-2.44233	-0.80956	0.39274	14.82357	-0.40945	0.59049	-0.99394
**Liver**	1.38087	9.37352	13.55239	7.87413	8.23663	30.78577	12.15000	-0.42266
**TemporalLobe**	-2.82147	-4.02738	-3.57148	-3.54484	9.75669	-0.42336	-3.26268	-0.99394
**TestisIntersitial**	-3.29720	-6.18882	-0.80956	-5.90739	-5.24125	-0.43819	-5.67092	-1.31683
**OccipitalLobe**	-2.82147	-4.60377	-4.40005	-3.54484	-3.51852	-0.43495	-3.74433	1.46502
**Tongue**	-2.18715	-1.72185	2.50473	-1.57605	-1.39043	-0.38350	-0.37281	-1.06845
**OlfactoryBulb**	-3.69365	-7.34158	-4.40005	-6.69491	-6.45730	-0.45349	-8.07915	-1.41618
**UterusCorpus**	-2.34573	-4.89196	-0.25718	-1.96981	-3.51852	-0.40528	-4.22598	-1.21748
**Bonemarrow**	-0.04634	-0.42499	3.33331	-1.96981	-0.88374	-0.38952	-0.37281	-1.04361
**Spinalcord**	-1.47355	-1.57775	0.29520	0.78649	-0.47839	-0.41084	2.99872	-0.91942
**DorsalRootGanglion**	-3.13863	-4.45967	-2.74290	-1.96981	-6.05195	-0.41733	-8.56079	-1.44102
**pineal_night**	74.35887	1.04479	4.54855	5.43283	3.37243	-0.35115	8.97113	-0.62137
**small_intestine**	24.29552	-3.73919	-18.48582	-1.96981	-10.30813	-0.47527	-1.33610	-1.52795
**Pons**	-1.31497	-3.45100	-1.08576	-1.18230	11.88478	-0.41501	-1.81775	-1.09329
**TestisSeminiferousTubule**	-2.82147	-5.90063	-6.05720	-5.11987	-4.73457	-0.42521	-5.67092	-1.29199
**PancreaticIslet**	-1.39426	25.36815	0.57139	1.18025	1.14301	-0.40296	2.51707	-0.89459
**TrigeminalGanglion**	-1.31497	1.44826	8.02856	4.33031	3.47377	-0.32417	9.74177	-1.01878
**SubthalamicNucleus**	-2.58360	-4.60377	1.12378	-1.96981	-1.79578	-0.41780	-3.26268	-0.05010
**CD33+_Myeloid**	0.03295	0.87187	7.75237	7.48038	3.57511	-0.37979	11.66835	-0.49718
**GlobusPallidus**	-4.09010	-8.20616	-7.71435	-8.66369	-1.79578	-0.44329	-10.00573	-1.46586
**Placenta**	9.62697	-2.87462	11.34286	0.39274	-3.01183	-0.42475	1.07213	-0.94426
**CingulateCortex**	-1.86999	-2.44233	-0.25718	0.39274	2.76441	-0.40992	1.07213	-0.86975
**CD4+_Tcells**	-1.39426	-3.30690	-0.80956	1.57401	-0.17438	-0.42428	3.48036	-0.86975

**ParietalLobe**	-1.07710	-1.00137	1.95235	0.78649	1.34568	-0.38767	2.03542	-0.77040
**Lung**	6.69326	3.03331	1.95235	1.96777	0.12963	-0.40528	3.96201	-0.86975
**CD8+_Tcells**	-2.74218	-5.61244	-4.40005	-1.96981	-2.70782	-0.44514	-0.85445	-1.06845
**pineal_day**	18.47567	1.56353	5.10093	5.90534	3.49404	-0.21284	9.74177	-0.56176
**Adrenalgland**	-3.21792	-5.18015	-5.50482	-5.90739	-4.43055	-0.42985	-5.67092	-1.31683
**Trachea**	-3.29720	2.74512	-6.88578	-5.51363	-5.64660	-0.44375	-6.63421	-1.26715
**SmoothMuscle**	-1.23568	-0.42499	2.78092	3.14904	1.24435	-0.40111	5.40695	-0.77040
**Lymphnode**	-3.21792	36.31943	-7.16197	-5.51363	-5.03858	-0.44700	-4.70762	-1.29199
**Hypothalamus**	-1.63213	-0.13680	1.12378	28.34954	0.83900	-0.41641	3.48036	-0.79523
**FetalThyroid**	-2.26644	-2.87462	-2.19052	0.39274	0.94033	-0.41362	-0.85445	-1.06845

Fluoxetine's HR profile of scores were calculated by combining target affinities and target-tissue expression levels according to the formula specified in the Methods. The left column lists all of the human tissues for which expression levels were measured, while the header lists fluoxetine's targets.

Supplemental Table 4b. Fluoxetine's HR scores for human data

	Muscarinic acetylcholine receptor M5	Potassium voltage-gated channel subfamily H member 2	5-HT receptor 2A	5-HT receptor 2C	5-HT receptor 6	Sigma non-opioid intracellular receptor 1	Cytochrome P450 2C19
**Prostate**	-0.497994	-0.942686	0.015443	-1.160986	1.585060	2.002298	0.999701
**Adipocyte**	-1.120337	-1.004289	-2.094316	-1.626906	-1.280375	7.011641	-2.585890
**Thymus**	-3.287013	-1.048096	-4.331940	-2.168674	-9.458804	-4.727901	-6.926341
**PrefrontalCortex**	0.447045	-0.931735	49.690690	-1.030962	0.361280	-1.203681	1.377131
**Cerebellum**	2.775070	-0.703804	-2.254146	-1.551059	2.256646	-2.288057	-2.680247
**colon**	-2.457223	-0.997444	-1.966452	-1.540223	-8.742445	-1.415842	-1.831029
**AtrioventricularNode**	3.143866	-1.052202	-2.669705	-1.594400	12.628924	-3.242778	21.758383
**Heart**	2.613721	2.494756	1.294085	-0.294159	14.389972	1.318670	5.906299
**CD71+_EarlyErythroid**	-1.696581	14.397806	-2.669705	-1.702754	-2.205672	9.970100	-3.340751
**Fetalbrain**	-1.120337	-0.915307	-1.838588	-1.659412	-0.862499	-2.181976	-2.208459
**CiliaryGanglion**	2.060528	-1.056309	-4.843397	-2.027814	3.853529	-3.490299	3.641715
**Thyroid**	0.066725	-0.978279	-0.751742	-1.307263	-0.578940	-0.938481	0.056124
**TestisGermCell**	-2.641621	1.777424	-3.628687	-2.071155	-5.041258	-3.372432	-5.794050
**CD19+_BCells(neg._sel.)**	-4.185954	-0.983755	-1.263199	-1.453541	-2.205672	3.086674	-1.076167
**SkeletalMuscle**	48.574915	-0.968696	-0.112421	-0.619219	20.001449	0.564322	5.717583
**Uterus**	1.611057	-0.964589	-3.500823	-1.821942	-4.787548	-4.951848	-3.529466
**Kidney**	-0.705442	-1.043989	-4.012279	-1.756930	-0.175989	-3.879259	-4.095612
**Appendix**	1.299886	-0.997444	-1.391063	-0.835926	1.316425	-2.653444	14.964633
**CD14+_Monocytes**	-4.139854	-0.976910	-1.263199	-1.410199	-3.638389	10.995542	-0.887452
**CerebellumPeduncles**	0.838891	-0.630565	-0.432081	-1.085139	6.838357	0.293228	0.810986
**BDCA4+_DentriticCells**	-3.517511	-0.987862	-1.071402	-1.453541	-10.443797	1.106510	-2.019744
**Pituitary**	2.452373	0.246934	0.015443	-1.150151	6.510026	-2.299843	1.377131
**retina**	-1.696581	-0.801000	0.367070	-1.025544	3.659515	1.300990	2.509423
**Testis**	-0.700832	-0.050402	-3.232308	-1.873952	-2.193732	-3.735462	-4.246584
**Amygdala**	-1.143387	-0.959114	-1.327131	10.562859	-5.071107	-3.973553	-1.076167
**Fetallung**	-2.595521	-1.035775	-3.820483	-2.060320	-5.011410	-4.445021	-6.737626
**TestisLeydigCell**	0.032150	-0.990600	-1.774656	-1.496882	1.107487	-2.016963	-0.321306
**Salivarygland**	-2.249775	-1.046727	-4.204076	-1.995308	-3.280210	-4.692541	-5.416619
**WholeBlood**	-0.958989	-0.994706	-1.007470	-1.366858	-0.743106	-3.949980	-0.321306
**CD34+**	-0.336646	3.495461	1.102289	-1.009291	-1.817644	17.713954	2.886854
**Caudatenucleus**	-1.834880	-0.967327	-3.117230	50.913690	-3.459299	-4.138567	-4.473043

**CardiacMyocytes**	0.539244	-0.942686	1.421950	-0.250817	18.150856	5.055051	7.038590
**CD105+_Endothelial**	-4.070705	40.372328	-1.774656	-1.572729	-5.488982	25.669969	-1.831029
**Tonsil**	-1.558283	-1.005658	-2.222180	-1.605236	3.375957	-2.700591	-2.208459
**SuperiorCervicalGanglion**	4.111955	-0.972803	1.102289	-0.413347	11.852868	-0.791147	7.227306
**CD56+_NKCells**	-3.125665	-0.953638	0.015443	-1.150151	-7.041093	6.976281	0.433555
**Fetalliver**	-2.065377	-0.445756	-3.500823	-1.843613	-4.503989	5.950839	-4.850473
**Skin**	-2.618571	-1.052202	-3.692619	-1.756930	-1.817644	-3.726033	14.775917
**Ovary**	-2.664670	-1.074106	-5.099125	-2.222850	-5.459134	-4.044273	-5.605334
**Wholebrain**	-3.102616	-0.746926	-3.117230	-1.941131	-9.667742	-3.372432	-5.794050
**Pancreas**	-2.572471	-1.041251	-4.140144	-1.951967	-4.742775	-3.372432	-5.794050
**BronchialEpithelialCells**	-1.996228	-1.015241	-2.158248	-1.637742	-5.011410	7.695269	-2.774605
**AdrenalCortex**	-0.451895	-0.985124	-1.199267	-1.117645	9.465006	2.461979	2.131993
**MedullaOblongata**	0.032150	-1.002920	5.002148	-0.554207	-1.101285	-3.949980	-4.095612
**Thalamus**	-1.489133	-0.852335	-1.774656	-1.366858	2.480508	-0.413973	-1.831029
**Liver**	2.728970	-0.907093	1.741610	-0.424183	6.360785	38.104926	53.085122
**TemporalLobe**	-1.673531	-1.019348	8.390549	-1.724424	2.211874	0.033921	-3.152036
**TestisIntersitial**	-2.318924	-1.017979	-3.756551	-1.941131	-2.295216	-4.421447	-5.227904
**OccipitalLobe**	-3.010417	-1.009765	21.688428	-1.854448	-0.862499	-2.040536	-4.661758
**Tongue**	-0.290547	-1.005658	-1.838588	-1.345187	7.584564	-3.207418	-0.321306
**OlfactoryBulb**	-1.904029	-0.987862	-4.268008	-2.103661	-6.324734	-2.488430	-6.548911
**UterusCorpus**	5.172243	-1.024823	-3.053298	-1.572729	-1.668403	-4.586461	-0.887452
**Bonemarrow**	3.143866	0.698689	-2.030384	-1.410199	-0.355078	-2.700591	-1.642313
**Spinalcord**	-0.659342	-0.927628	-1.199267	-1.399364	0.032949	-0.355040	-1.076167
**DorsalRootGanglion**	-2.272825	-1.043989	-2.925434	-1.713589	2.062632	-4.350727	-4.850473
**pineal_night**	-0.216787	0.433112	0.475755	24.529621	-3.053363	0.154145	2.773625
**small_intestine**	-2.019277	-1.074106	-5.546650	-2.407051	-7.608210	1.271523	-2.963320
**Pons**	6.393880	-0.981017	1.869474	-1.583565	-0.474471	-2.523790	-2.585890
**TestisSeminiferousTubule**	-0.382746	-0.749664	-3.500823	-1.930296	-0.116292	-3.914620	-5.039189
**PancreaticIslet**	-0.567143	-0.844122	-1.199267	-1.366858	1.137336	-2.370563	-0.132591
**TrigeminalGanglion**	-0.820691	-1.005658	-1.454995	-1.085139	15.136179	-1.840162	14.209771
**SubthalamicNucleus**	1.115488	-0.937210	18.236094	-1.702754	1.137336	-3.525659	-3.152036
**CD33+_Myeloid**	-2.687720	-0.937210	1.230153	-0.955115	-2.026582	-1.910882	3.453000
**GlobusPallidus**	3.512662	-1.059047	2.253067	-2.114497	3.316260	-3.832113	-6.926341
**Placenta**	-1.973178	-1.009765	-1.582859	-1.572729	-4.742775	3.051313	-2.208459
**CingulateCortex**	1.230737	-0.934473	11.331426	-1.442705	7.674109	-1.486562	-1.642313

**CD4+_Tcells**	-4.324252	-0.972803	-1.071402	-1.442705	-3.310058	-0.013226	-0.887452
**ParietalLobe**	3.443512	-0.987862	10.244580	-1.301846	3.017777	-2.111256	0.433555
**Lung**	0.308747	-0.967327	-0.815674	-1.334352	-0.743106	-2.346990	-0.132591
**CD8+_Tcells**	-3.748008	-0.997444	-2.413977	-1.778601	-8.324569	-2.877391	-3.340751
**pineal_day**	-0.493384	0.631336	0.782629	-0.963783	-0.235685	1.507257	2.698139
**Adrenalgland**	-1.327785	-1.035775	-3.628687	-1.865284	-2.474306	-1.887309	-4.473043
**Trachea**	-1.419984	-1.038513	-3.756551	-2.006143	-4.384596	-4.798621	-5.605334
**SmoothMuscle**	-0.982039	-0.974172	-0.559946	-1.247669	0.629915	0.776483	0.244840
**Lymphnode**	-2.180626	-1.030299	-3.436890	-2.006143	-5.578527	-4.503954	-5.227904
**Hypothalamus**	-0.198348	-0.937210	-0.751742	22.730953	0.570218	-2.170190	-0.698737
**FetalThyroid**	0.423996	-1.009765	-2.222180	-1.605236	-0.295382	-1.840162	-2.774605

Fluoxetine’s HR profile of scores were calculated by combining target affinities and target-tissue expression levels according to the formula specified in the Methods. The left column lists all of the human tissues for which expression levels were measured, while the header lists fluoxetine’s targets.

Supplemental Table 5. Venlafaxine's HR scores for human data

	Sodium-dependent noradrenaline transporter	Sodium-dependent serotonin transporter	Sodium-dependent dopamine transporter
**Skin**	-3.325350174	-3.459774023	-7.490777632
**Pituitary**	3.642429389	-0.529205875	4.118420009
**Hypothalamus**	1.134028746	-1.436286492	26.96232504
**Bonemarrow**	3.363718206	-0.04077785	-1.873423934
**CD56+_NKCells**	4.757274119	-0.598981307	4.492910256
**CD4+_Tcells**	-0.816949531	-1.226960196	1.496988284
**Cerebellum**	2.527584659	2.087372829	-0.188217825
**TestisLeydigCell**	0.576606381	-1.924714517	-1.498933688
**BDCA4+_DentriticCells**	-1.653083079	-1.785163653	1.87147853
**OlfactoryBulb**	-4.440194904	-3.250447727	-6.367306892
**Tongue**	2.527584659	-1.924714517	-1.498933688
**Adipocyte**	-2.210505444	-1.924714517	2.99494927
**CD19+_BCells(neg._sel.)**	0.855317564	-1.436286492	0.748007791
**UterusCorpus**	-0.259527166	-2.064265381	-1.873423934
**TestisIntersitial**	-0.816949531	-2.901570566	-5.618326399
**CD8+_Tcells**	-4.440194904	-2.413142541	-1.873423934
**CingulateCortex**	-0.259527166	-1.645612788	0.373517545
**Uterus**	-3.185994582	-2.622468838	-3.93312029
**DorsalRootGanglion**	-2.767927809	-2.762019702	-1.873423934
**Trachea**	-6.948595546	-2.901570566	-5.243836153
**Prostate**	2.806295841	-0.808307603	3.743929763
**FetalThyroid**	-2.210505444	-1.994489949	0.373517545
**AdrenalCortex**	4.478562936	-1.36651106	2.245968777
**Pons**	-1.095660714	-1.157184764	-1.124443441
**retina**	5.035985301	-0.494318159	4.680155379
**PrefrontalCortex**	4.757274119	-0.180328714	5.616380995
**CD14+_Monocytes**	0.576606381	-1.575837356	1.122498038
**TestisSeminiferousTubule**	-6.112461999	-2.482917974	-4.869345906
**WholeBlood**	2.527584659	-1.296735628	1.496988284
**Lung**	1.970162294	5.890133879	1.87147853
**CD33+_Myeloid**	7.823097127	0.028997582	7.114341981
**Spinalcord**	0.297895199	-1.296735628	0.748007791
**Thyroid**	1.134028746	-1.157184764	3.182194393
**Amygdala**	-0.259527166	-1.436286492	0.373517545
**SubthalamicNucleus**	1.134028746	-2.273591677	-1.873423934

**Tonsil**	-2.210505444	-1.994489949	-1.873423934
**colon**	-1.653083079	-1.785163653	-1.124443441
**Fetalliver**	-4.718906086	0.656976471	-5.243836153
**Thalamus**	-0.816949531	-1.71538822	0.373517545
**Heart**	11.4463425	0.308099311	5.616380995
**Appendix**	5.314696484	-1.575837356	-0.375462948
**CD71+_EarlyErythroid**	-3.325350174	-2.203816245	-2.996894674
**Thymus**	-8.063440276	-3.320223159	-4.120365413
**TrigeminalGanglion**	8.101808309	-1.157184764	4.118420009
**OccipitalLobe**	-4.440194904	-2.482917974	-3.37138492
**pineal_night**	4.590047409	65.43648764	5.1669927
**Wholebrain**	-6.948595546	-2.762019702	-4.869345906
**Caudatenucleus**	-4.718906086	-0.947858467	-3.745875167
**ParietalLobe**	1.970162294	-0.947858467	0.748007791
**AtrioventricularNode**	-1.374371896	-3.459774023	-4.869345906
**Kidney**	-6.391173181	-2.971345998	-6.367306892
**small_intestine**	-18.65446521	21.38027981	-1.873423934
**Liver**	13.67603196	1.215179928	7.488832228
**SuperiorCervicalGanglion**	13.95474314	5.61103215	2.245968777
**TemporalLobe**	-3.604061356	-2.482917974	-3.37138492
**pineal_day**	5.147469774	16.25876309	5.616380995
**SmoothMuscle**	2.806295841	-1.087409331	2.99494927
**CD34+**	6.986963579	0.028997582	7.114341981
**CardiacMyocytes**	10.61020895	-0.180328714	5.241890749
**PancreaticIslet**	0.576606381	-1.226960196	1.122498038
**CD105+_Endothelial**	-1.653083079	-1.924714517	-0.749953195
**GlobusPallidus**	-7.784729094	-3.599324887	-8.239758125
**Fetalbrain**	-1.931794261	-1.506061924	-1.498933688
**Ovary**	-7.506017911	-4.087752912	-10.4866996
**CerebellumPeduncles**	13.11860959	-0.878083035	5.990871242
**Salivarygland**	-8.063440276	-3.180672295	-5.618326399
**Adrenalgland**	-5.555039634	-2.831795134	-5.618326399
**Pancreas**	-6.391173181	-3.110896863	-4.49485566
**Lymphnode**	-7.227306729	-2.831795134	-5.243836153
**Testis**	-2.099020971	-2.622468838	-4.345059561
**Placenta**	11.4463425	8.471824867	0.373517545
**TestisGermCell**	-6.948595546	-3.110896863	-4.869345906
**Fetallung**	-8.899573824	-2.901570566	-5.243836153
**MedullaOblongata**	-4.440194904	-1.924714517	-3.37138492
**BronchialEpithelialCells**	-3.325350174	-1.994489949	-1.498933688

**CiliaryGanglion**	-5.555039634	-3.738875751	7.863322474
**SkeletalMuscle**	23.43092335	-0.459430443	14.97863716

Venlafaxine's HR profile of scores were calculated by combining target affinities and target-tissue expression levels according to the formula specified in the Methods. The left column lists all of the human tissues for which expression levels were measured, while the header lists venlafaxine's targets.

Supplemental Table 6. Bupropion's HR scores for rat data

	Sodium-dependent dopamine transporter	Sodium-dependent noradrenaline transporter	Aldo-keto reductase family 1 member C3	Neuronal acetylcholine receptor subunit alpha-3	5-HT receptor 3A
**Nucleus accumbens_core**	-1.138709216	0.599739577	1.550533765	-1.597785768	8.182159871
**Frontal cortex**	-1.185456411	-1.668602135	-1.319553215	-1.69627572	-3.087046736
**Cerebral cortex**	-1.282691499	-2.35373392	-3.31307081	-1.712861064	-1.838789244
**Hippocampus**	-1.266787812	-1.55108993	-2.11659878	-1.715927256	-3.827472763
**Dorsal root ganglion**	-1.141141814	0.501456643	1.37521989	-1.60345359	2.570816025
**Primary cortical neurons**	-1.11302924	0.716539592	2.056594445	-1.592396694	2.198664724
**Skeletal muscle**	-1.175565378	-0.163733676	0.37755741	-1.641270015	1.18687837
**Small intestine**	-1.242599524	-1.905050942	-2.760018785	-1.736136279	-5.506030199
**Amygdala, central nucleus**	-1.162943404	-0.676514219	0.01427814	-1.649957571	-1.061535744
**Large intestine**	-1.317780578	-2.63362663	-4.61979177	-1.750398741	-5.149385198
**Amygdala**	-1.133362092	-0.263441003	1.606561915	-1.699481295	4.350164435
**Cerebellum**	-1.318239564	-2.216280243	-3.285960415	-1.704870369	-3.32739445
**Spleen**	-1.220797934	-1.877275331	-2.705797995	-1.715880798	-2.554017529
**Bone marrow**	-1.193029596	-0.988455721	-0.173687255	-1.660410513	-0.803743437
**Prefrontal cortex**	-1.17042479	0.74930057	0.579981685	-1.644614955	1.572597688
**Thymus**	-1.266397676	-2.106602183	-1.885256755	-1.745195499	-2.784673803
**Hypothalamus**	-1.224538626	-1.131606955	0.363098535	-1.682617212	-2.470671141
**Pineal**	-1.212972312	-1.460641135	-1.82742125	48.46577195	-3.965091214
**Nucleus accumbens_shell**	-1.158812575	0.198773683	-0.02548377	-1.643221233	1.71990758
**Pituitary**	-1.245376358	-2.276816838	-3.23173963	-1.720665927	-3.941831757
**Heart**	-1.20512374	-1.054689874	-1.04664192	-1.681780977	-1.753504571
**Ventral tegmental area**	34.22048425	-0.160172702	0.42635612	-1.630027296	1.615240026
**Ventral striatum**	-1.179627357	-1.143714271	0.59444056	-1.635927399	10.7523298
**Dorsal raphe**	-0.699395728	-1.075343536	0.46792539	-1.692744948	-2.422213942
**Cornea**	-1.160441957	-0.066875128	25.03536389	-1.619481438	1.969946735
**Locus coeruleus**	-1.215657352	27.25791813	-0.7122804	-1.673325702	1.2954225
**Nucleus accumbens, whole**	-1.009184832	-0.036250738	0.766139715	-1.61943498	6.511355587
**Endothelial cells**	-1.260660422	-1.962026557	-4.140841485	-1.720340721	-2.823439565
**Kidney**	-1.247304076	-1.130182563	-2.98413137	-1.717320978	-3.434000292
**Dorsal striatum**	-1.072432386	-0.121001965	0.934224155	-1.561967019	6.825358249

Bupropion's HR profile of scores were calculated by combining target affinities and target-tissue expression levels according to the formula specified in the Methods. The left column lists all of the rat tissues for which expression levels were measured, while the header lists bupropion's targets.

Supplemental Table 7. Duloxetine's HR scores for rat data

	Sodium-dependent serotonin transporter	Sodium-dependent noradrenaline transporter	Sodium-dependent dopamine transporter	Cytochrome P450 2B6
**Nucleus accumbens_core**	-1.92803179	0.956466225	-1.186050135	1.684912155
**Frontal cortex**	-2.335429498	-2.661090989	-1.234740806	-3.115128285
**Cerebral cortex**	-2.446493576	-3.753740928	-1.336018365	-3.80924492
**Hippocampus**	-2.261224044	-2.473682221	-1.319453495	-3.88761293
**Dorsal root ganglion**	-2.173837354	0.799724347	-1.188583866	7.1286898
**Primary cortical neurons**	-1.66806861	1.14273919	-1.159302534	0.811668645
**Skeletal muscle**	-2.187262683	-0.261122889	-1.224438562	1.39103213
**Small intestine**	3.181648299	-3.038180157	-1.294259599	-4.26265982
**Amygdala, central nucleus**	-2.184089422	-1.078906621	-1.21129184	0.338661745
**Large intestine**	-1.134961013	-4.200114543	-1.372566245	-6.540929745
**Amygdala**	-2.180427967	-0.420136391	-1.180480708	-0.027988575
**Cerebellum**	-2.305893774	-3.53452945	-1.373044313	-3.708486055
**Spleen**	-2.062040979	-2.99388354	-1.271551625	-4.427792405
**Bone marrow**	-0.305519797	-1.576391734	-1.242628841	-1.41062413
**Prefrontal cortex**	-2.155041893	1.194986482	-1.219084258	2.793259685
**Thymus**	-2.352760374	-3.359614597	-1.319047139	-3.288657445
**Hypothalamus**	-2.296373992	-1.804689692	-1.275447834	18.00784852
**Pineal**	-2.334209006	-2.32943425	-1.263400659	-4.29344725
**Nucleus accumbens_shell**	-1.897031492	0.317004782	-1.206989275	-0.640938345
**Pituitary**	-2.401823846	-3.631073368	-1.297151877	-4.987563885
**Heart**	-2.269523334	-1.682022131	-1.255225789	-2.933202555
**Ventral tegmental area**	-0.101698896	-0.255443838	35.64317333	0.039184005
**Ventral striatum**	-2.220703955	-1.82399847	-1.228669414	3.179502005
**Dorsal raphe**	51.47939025	-1.714960645	-0.728472542	9.563695735
**Cornea**	-1.842109693	-0.106652627	-1.208686397	0.69131778
**Locus coeruleus**	-1.338049626	43.47099816	-1.266197328	4.97636846
**Nucleus accumbens, whole**	-1.703950855	-0.05781277	-1.051140879	0.814467505
**Endothelial cells**	-2.390107194	-3.129045015	-1.313071363	-4.234671245
**Kidney**	-2.14845128	-1.802418068	-1.299159738	-2.871627695
**Dorsal striatum**	-2.035922617	-0.192974246	-1.117017899	3.019967135

Duloxetine's HR profile of scores were calculated by combining target affinities and target-tissue expression levels according to the formula specified in the Methods. The left column lists all of the rat tissues for which expression levels were measured, while the header lists duloxetine's targets.

Supplemental Table 8a. Fluoxetine's HR scores for rat data

	Sodium-dependent serotonin transporter	Alpha-2A adrenergic receptor	Sodium-dependent noradrenaline transporter	Sodium-dependent dopamine transporter	Alpha-2B adrenergic receptor	Muscarinic acetylcholine receptor M1	Muscarinic acetylcholine receptor M3
**Nucleus accumbens_core**	-1.917457837	3.912803139	0.73927576	-1.025060665	10.10191749	4.450871676	3.569083298
**Frontal cortex**	-2.322621243	-2.653636426	-2.056821256	-1.067142269	-4.141984754	-0.039238686	11.63851845
**Cerebral cortex**	-2.43307621	-2.617688764	-2.901356685	-1.154672836	-6.254198399	3.789447243	9.740712154
**Hippocampus**	-2.248822756	-12.13782789	-1.91196851	-1.140356412	-6.362517043	-3.614427148	17.97909573
**Dorsal root ganglion**	-2.161915323	-8.474162013	0.618126191	-1.027250478	7.213420202	0.534772825	-3.436549459
**Primary cortical neurons**	-1.658920379	21.76081727	0.883250616	-1.001943671	11.36563505	14.52958144	2.778032386
**Skeletal muscle**	-2.175267023	1.18078084	-0.201828164	-1.058238407	6.112180612	2.524873656	-0.012480231
**Small intestine**	3.164199105	-5.15199893	-2.348282547	-1.118582229	-6.579154338	-9.07190717	-4.120217889
**Amygdala, central nucleus**	-2.172111166	-10.73586908	-0.833912888	-1.046876166	1.725275358	-5.438443428	1.17373819
**Large intestine**	-1.128736518	-7.335819392	-3.246369592	-1.18625986	-11.14659067	-11.30093665	-8.372563955
**Amygdala**	-2.168469791	-3.639201492	-0.324733526	-1.020247208	-4.737737323	3.046437414	6.197090434
**Cerebellum**	-2.293247502	-2.659627702	-2.731922858	-1.186673037	-6.434729474	-4.258368997	-6.115384303
**Spleen**	-2.050732076	-3.120956033	-2.314044626	-1.098956541	-3.383754219	-3.553238103	-6.538471259
**Bone marrow**	-0.303844227	12.94465319	-1.218431102	-1.073959614	3.476426838	6.16999245	-5.267062738
**Prefrontal cortex**	-2.143222943	2.711552106	0.923633805	-1.053610874	2.375187248	12.51034297	1.640494019
**Thymus**	-2.339857071	-5.849982694	-2.59672696	-1.140005213	-5.351542994	-7.396492857	-8.18428668
**Hypothalamus**	-2.283779931	-4.498949742	-1.394888086	-1.102323895	-1.885346247	-4.51478023	-2.978384228
**Pineal**	-2.321407445	-4.07956035	-1.800475782	-1.091911953	-4.196144076	-3.436687545	-3.049972543
**Nucleus accumbens_shell**	-1.886627554	9.379843383	0.245020624	-1.043157614	4.433241564	6.580833176	2.968457314
**Pituitary**	-2.388651463	-6.874491061	-2.806543976	-1.121081922	-6.20003907	-6.915721787	-5.461067078
**Heart**	-2.25707653	-1.212734314	-1.300075378	-1.084846706	-0.188354095	-0.76476593	-2.165856823
**Ventral tegmental area**	-0.101141147	6.800598649	-0.197438689	30.80511848	5.606693585	2.74631972	3.265548828
**Ventral striatum**	-2.208524892	-3.366598388	-1.409812306	-1.061894982	1.400319412	-1.796238395	0.523716267
**Dorsal raphe**	51.19706054	-3.330650725	-1.325534348	-0.629592735	-3.636497727	-0.834696268	6.24505461
**Cornea**	-1.832006965	19.35831519	-0.082434382	-1.044624375	7.70085412	1.81391523	2.503849132
**Locus coeruleus**	-1.330711327	13.13337841	33.59978046	-1.094329014	-1.415965438	-0.327701328	-3.322724033
**Nucleus accumbens, whole**	-1.694605834	6.081645411	-0.044684881	-0.908463425	7.321738852	1.776036301	1.346981923
**Endothelial cells**	-2.376999069	-3.501402125	-2.418514183	-1.134840564	-3.744816377	-4.698347366	-9.512965853
**Kidney**	-2.136668474	-4.06458216	-1.393132294	-1.122817245	-5.225171234	-4.473987535	-3.385005872
**Dorsal striatum**	-2.024756956	-1.9586483	-0.149154438	-0.965398575	12.05165316	11.96255533	0.352620188

Fluoxetine’s HR profile of scores were calculated by combining target affinities and target-tissue expression levels according to the formula specified in the Methods. The left column lists all of the rat tissues for which expression levels were measured, while the header lists fluoxetine’s targets.

Supplemental Table 8b. Fluoxetine's HR scores for rat data

	Muscarinic acetylcholine receptor M5	Potassium voltage-gated channel subfamily H member 2	5-HT receptor 2A	5-HT receptor 2C	5-HT receptor 6	Sigma non-opioid intracellular receptor 1
**Nucleus accumbens_core**	2.526643758	-1.115375066	7.51382281	23.96052986	5.084178203	-1.886439039
**Frontal cortex**	-4.450954187	-0.525855049	0.801739339	-3.518743753	-2.984561457	-1.194805439
**Cerebral cortex**	-3.117572761	-3.304107923	-8.068816936	-4.798566615	-3.778137658	-1.045083298
**Hippocampus**	-3.476346684	-3.673157037	-5.1083126	-4.538602598	-3.803875262	-1.143579976
**Dorsal root ganglion**	-2.518845665	-2.154623767	8.804523804	1.637465669	3.209622528	-0.805132454
**Primary cortical neurons**	4.536887323	1.715599108	8.072615258	1.171376337	6.315293719	-2.171144764
**Skeletal muscle**	6.395021841	-2.08752393	1.818522471	-1.129843625	0.738812295	-7.584507779
**Small intestine**	-5.751971986	-3.485437249	-7.66341849	-5.450784042	-7.334216966	-1.915376935
**Amygdala, central nucleus**	-3.412544105	-4.694831975	1.119484067	-1.419034364	0.309852187	-0.652893972
**Large intestine**	-4.881390428	-3.976703932	-11.70206355	-6.938270468	-11.89835253	0.018968396
**Amygdala**	-3.223910393	-1.705693892	-1.111303058	-2.12662874	-0.535199227	-0.539479005
**Cerebellum**	-1.717614736	0.21224318	-4.948344568	-4.570905823	-3.881088086	-1.758644994
**Spleen**	-8.384521867	-2.425419548	-6.260958995	-4.281715077	-2.160958046	-2.61527851
**Bone marrow**	-2.983494883	-0.502689632	5.484639233	-2.385054508	0.001000909	-3.106144246
**Prefrontal cortex**	14.96953361	12.63609796	3.834557988	-1.248288768	7.992527746	-0.626831896
**Thymus**	-6.574008109	2.681357511	-4.247114821	-4.537064351	-2.645682969	-2.051079476
**Hypothalamus**	4.494814608	-0.926057664	-3.499866879	-3.164946565	-0.247795959	-1.140344685
**Pineal**	-5.233229283	-3.503809826	-6.346421369	-4.069436763	-1.710549929	-1.659069888
**Nucleus accumbens_shell**	6.454663382	-1.303094854	1.073465865	3.807934499	1.184930812	-1.607844426
**Pituitary**	-4.600751547	2.854698762	-7.29308153	-4.217108635	-2.834425416	-6.505537789
**Heart**	-2.621484596	1.237113349	-3.942518153	-3.352612901	-4.228545775	-3.541470807
**Ventral tegmental area**	5.297432555	5.798304807	2.585492503	3.447984313	22.3112162	0.290013999
**Ventral striatum**	-0.284368403	-4.630128555	22.31196177	2.349674788	1.013346767	-1.01308985
**Dorsal raphe**	13.14376271	2.976117517	-3.775976091	-3.321847931	-1.427436255	7.201497096
**Cornea**	2.443423003	-0.600144157	0.674641449	-0.480702701	6.564090582	-2.884167249
**Locus coeruleus**	11.56719175	19.32211767	0.389766864	7.593564391	-1.071399364	11.02741002
**Nucleus accumbens, whole**	3.09809294	-1.450075457	16.43039729	21.96849788	2.613367976	-3.043235786
**Endothelial cells**	-7.023862527	-3.07165491	-3.852673092	-4.478610899	-7.278452152	31.33551968
**Kidney**	-4.760720332	-3.141949978	-5.467692851	-5.169284535	-6.283264696	1.569751854
**Dorsal striatum**	0.090124996	-1.155315446	2.372932239	9.261025906	6.765701837	-0.951978775

Fluoxetine's HR profile of scores were calculated by combining target affinities and target-tissue expression levels according to the formula specified in the Methods. The left column lists all of the rat tissues for which expression levels were measured, while the header lists fluoxetine's targets.

Supplemental Table 9. Venlafaxine's HR scores for rat data

	Sodium-dependent noradrenaline transporter	Sodium-dependent serotonin transporter	Sodium-dependent dopamine transporter
**Nucleus accumbens_core**	0.746020308	-1.687380534	-0.974901869
**Frontal cortex**	-2.075586013	-2.043928058	-1.014924315
**Cerebral cortex**	-2.927826294	-2.141129445	-1.098171791
**Hippocampus**	-1.929411749	-1.978984711	-1.084555906
**Dorsal root ganglion**	0.623765469	-1.90250537	-0.976984529
**Primary cortical neurons**	0.891308673	-1.459865193	-0.952916048
**Skeletal muscle**	-0.20366948	-1.914254989	-1.006456141
**Small intestine**	-2.36970636	2.784524318	-1.063847189
**Amygdala, central nucleus**	-0.841520828	-1.911477806	-0.995649884
**Large intestine**	-3.275986818	-0.993298518	-1.128213183
**Amygdala**	-0.32769613	-1.908273363	-0.970323948
**Cerebellum**	-2.756846692	-2.018078896	-1.128606142
**Spleen**	-2.33515608	-1.80466309	-1.045181834
**Bone marrow**	-1.229547072	-0.267385714	-1.02140807
**Prefrontal cortex**	0.932060286	-1.886055903	-1.002055045
**Thymus**	-2.620417376	-2.059095746	-1.084221892
**Hypothalamus**	-1.407613906	-2.009747346	-1.048384415
**Pineal**	-1.816901853	-2.042859905	-1.038481956
**Nucleus accumbens_shell**	0.247255992	-1.660249601	-0.992113289
**Pituitary**	-2.832148591	-2.102035259	-1.066224565
**Heart**	-1.311936204	-1.986248108	-1.031762429
**Ventral tegmental area**	-0.199239958	-0.089005139	29.29774659
**Ventral striatum**	-1.422674282	-1.94352222	-1.009933791
**Dorsal raphe**	-1.337627441	45.0538842	-0.59878518
**Cornea**	-0.083186446	-1.61218298	-0.993508278
**Locus coeruleus**	33.90631742	-1.171038208	-1.040780744
**Nucleus accumbens, whole**	-0.04509255	-1.491268722	-0.864010026
**Endothelial cells**	-2.440578731	-2.091781045	-1.079309962
**Kidney**	-1.405842095	-1.880287911	-1.067874974
**Dorsal striatum**	-0.150515201	-1.781804745	-0.918159195

Venlafaxine's HR profile of scores were calculated by combining target affinities and target-tissue expression levels according to the formula specified in the Methods. The left column lists all of the rat tissues for which expression levels were measured, while the header lists venlafaxine's targets.
